# A randomized, double-blind, placebo-controlled crossover clinical trial to evaluate the anti-diabetic effects of *Allium hookeri* extract in the subjects with prediabetes

**DOI:** 10.1186/s12906-020-03005-3

**Published:** 2020-07-06

**Authors:** Soo-Hyun Park, Ui-Jin Bae, Eun-Kyung Choi, Su-Jin Jung, Sung-Hyen Lee, Jae-Heon Yang, You-Suk Kim, Do-Youn Jeong, Hyun-Ju Kim, Byung-Hyun Park, Soo-Wan Chae

**Affiliations:** 1 Clinical Trial Center for Functional Foods, Chonbuk National University Hospital, Jeonju, Republic of Korea; 2 Present address: Korea Food Research Institute, Wanju, Republic of Korea; 3 Department of Biochemistry and Molecular Biology, Chonbuk National University Medical School, Jeonju, Republic of Korea; 4 Department of Agro-food Resources, National Institute of Agricultural Sciences, Rural Development Administration, Wanju, Republic of Korea; 5 Center for Healthcare Technology Development, Chonbuk National University, Jeonju, Republic of Korea; 6 Goldtree Co., Ltd., Sunchang, Republic of Korea; 7 Microbial Institute for Fermentation Industry, Sunchang, Republic of Korea; 8 Research Department, World Institute of Kimchi, Gwangju, Republic of Korea; 9 Department of Pharmacology, Chonbuk National University Medical School, 567 Baekje-daero, Deokjin-gu, Jeonju-si, 54896 Republic of Korea

**Keywords:** *Allium hookeri*, Prediabetes, Plasma glucose, Oral glucose tolerance test, Hemoglobin A1c, Incremental area under the curve

## Abstract

**Background:**

*Allium hookeri* is widely consumed as a vegetable and herbal medicine in Asia. *A. hookeri* has been reported anti-inflammatory, anti-obesity, osteoblastic, anti-oxidant, and anti-diabetic effects in animal studies. We investigated the anti-diabetic effects of *A. hookeri* aqueous extract (AHE) in the Korean subjects.

**Methods:**

Prediabetic subjects (100 ≤ fasting plasma glucose (FPG) < 126 mg/dL) who met the inclusion criteria were recruited for this study. The enrolled subjects (*n* = 30) were randomly divided into either an AHE (*n* = 15, 486 mg/day) or placebo (n = 15) group. Outcomes were measurements of FPG, glycemic response to an oral glucose tolerance test (OGTT), insulin, C-peptide, hemoglobin A1c (HbA1c), total cholesterol, triglyceride, HDL-cholesterol, and LDL-cholesterol. The *t*-test was used to assess differences between the groups. A *p*-value < 0.05 was considered statistically significant.

**Results:**

Eight weeks after AHE supplementation, HbA1c level was significantly decreased in the AHE group compared with the placebo group. No clinically significant changes in any safety parameter were observed.

**Conclusion:**

The findings suggest that AHE can be effective in reducing HbA1c, indicating it as an adjunctive tool for improving glycemic control.

**Trial registration:**

The study protocol was retrospectively registered at www.clinicaltrials.gov (NCT03330366, October 30, 2017).

## Background

Type 2 diabetes mellitus is currently one of the greatest public health challenges, and the prevalence is rapidly increasing. In 2013, 382 million people globally were estimated to be diabetic, and this number is projected to reach 592 million by 2035 [1]. The effectiveness of lifestyle intervention, such as diet and exercise, for long-term maintenance of euglycemia in diabetic patients has been reported in systematic reviews and meta-analyses [2]. Guidelines suggested by the American Diabetes Association recommend changes in these lifestyle characteristics for both prevention and management of the disease [3]. Pharmacotherapy has also been used to manage patients with diabetes. Paradoxically, medications often cause metabolic side effects such as weight gain [4]. Thus, development of alternative therapies is important, and herbal extracts are among the most promising sources of new treatments for management of diabetes.

*Allium hookeri* Thwaites (Liliaceae family) is a traditional herb in Southeast Asia and is mainly used as a supplement and medicinal food [5]. *A. hookeri* was listed on the Plant List based on database of World Checklist of Selected Plant Families in 2012 (http://www.theplantlist.org). *A. hookeri* has been reported to have anti-inflammatory [6], anti-obesity [7], osteoblastic [8], anti-oxidant [9], and anti-diabetic [9] activities. However, these effects were observed in animal studies. Therefore, in this study, we investigated whether *A. hookeri* extract (AHE) could be effective in lowering blood glucose level in subjects with prediabetes.

## Materials and methods

### Preparation of AHE

Fresh *A. hookeri* root (100 kg) was purchased from the Hanam farm located in Sunchang-gun (Jeonbuk, Republic of Korea). *A. hookeri* was harvested on November 2014 and identified by the Agricultural Technology Research Institute. A voucher specimen (RDAAH15) has been kept in the Rural Development Administration. Root of *A. hookeri* were hot air-dried for 12 h at 60 °C. The dried roots (12 kg) were extracted with distilled water at 100 °C for 4 h. *A. hookeri* root extract (AHE) was concentrated under reduced pressure at 60 °C for 12 h to obtain concentrated one (7.2 kg). AHE was freeze dried at − 66 °C for 72 h under approximately 5–10 mmHg in a vacuum freeze-drying apparatus (4.5 kg). For standardization, the marker compound was cycloallin, and the sample was extracted with methanol and analyzed using LC-MS/MS under following condition: Column Acquity BEH Amide 1.7 μm (2.1 × 150 mm); mobile phase, solvent A: 10 mM CH_5_NO_2_, solvent B: 0.1% formic acid; flow rate 0.2 mL/min; temperature 25 °C. Standardized AHE containing 0.65–0.98 mg/g cycloallin was encapsulated to contain 243 mg of AHE and diluting ingredients. The placebo contains microstallin cellulose, hydroxypropyl metylcellulose, garlic flavor, and caramel flavor, so the flavor and appearance were not distinguished from AHE (Table 1).

**Table 1:** Composition of investigational products

Component	Content (%)	
	Placebo supplement	Test supplement
AHE	–	60.75
Microcrystalline cellulose	95.00	37.32
HPMC	2.94	1.15
Silica gel	–	0.75
Garlic flavor	2.00	–
Caramel flavor	0.06	0.03

*AHE Allium hookeri* extract; *HPMC* hydroxypropyl methylcellulose

### Study design

**Fig. 1:**
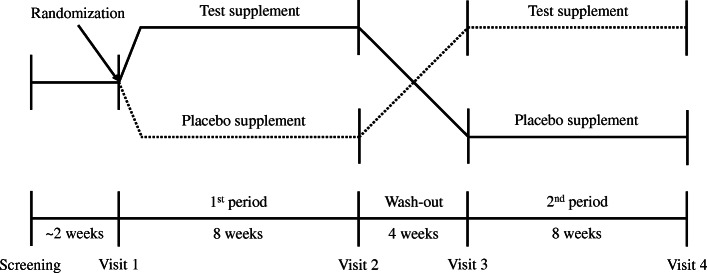
Scheme of the crossover design

The study was designed as an eight-week, randomized, double-blind, and placebo-controlled crossover trial. Random allocation sequence was generated using a computer program, and was concealed from all subjects, investigators, and staff until the end of the study. The trial protocol and informed consent form were approved by the Institutional Review Board of Chonbuk National University Hospital (IRB No.: 2015–02-038) and trial was conducted from 2015 to 2017. This trial adhered to CONSORT guidelines and included CONSORT checklist as an Additional file 1. Prior to the trial, written consent was obtained from all subjects, and the entire process of the trial was conducted in accordance with the Helsinki Declaration, International Conference on Harmonization (ICH-GCP), and trial protocol. Subjects were randomly assigned to receive either AHE or placebo in the first period. Based on the crossover design, subjects received the opposite investigational product (IP) after a four-week washout period (Fig. 1). During the trial period, subjects were instructed to take the IP twice daily and maintained their usual diet, except for taking functional foods or dietary supplements. Compliance was calculated by collecting the remaining IP consumed for 8 weeks from the prescribed IP, and subjects with compliance less than 70% were excluded from the analysis. The subjects also reported to the researchers the adverse events and lifestyle changes. Efficacy and safety were assessed before and after taking the investigational products.

### Subjects

The study subjects were recruited from the Clinical Trial Center for Functional Foods at Chonbuk National University Hospital. Prediabetes subjects (100 ≤ fasting plasma glucose (FPG) < 126 mg/dL) who had not been diagnosed with any disease and met the inclusion criteria were recruited for this study. Exclusion criteria for the study were as follows: (1) significant variation in body weight (> 10%) in the past 3 months; (2) diabetes; (3) total cholesterol ≥260 mg/dL and/or LDL-cholesterol ≥180 mg/dL; (4) familial combined hyperlipidemia; (5) systolic blood pressure > 160 mmHg and/or diastolic blood pressure > 100 mmHg; (6) use of corticosteroids within the past 4 weeks; (7) use of anti-obesity, hypolipidemic, or hypoglycemic drugs within the past 6 weeks; (8) use of obesity-, blood lipid-, or blood glucose-related functional foods within the past 2 weeks; (9) cardiovascular disease, heart failure, or myocardial infarction; (10) local or systemic inflammatory disease such as rheumatoid arthritis or autoimmune disease; (11) history of alcohol or substance abuse; (12) history of a disease that could interfere with the test products or impede their absorption, such as gastrointestinal disease or gastrointestinal surgery; (13) allergic or hypersensitivity; (14) participation in any other clinical trials within the past 2 months; (15) a laboratory test or medical or psychological conditions deemed by the investigators to interfere with successful participation in the study; (16) pregnancy or breast feeding.

### Efficacy outcome measures

At every visit, all subjects underwent blood tests and oral glucose tolerance test (OGTT) after an overnight fast. After a fasting blood sample was collected, glucose load of 75  g was ingested within 5 min. Blood samples were collected at 30, 60, 90, and 120 min after the glucose load. Blood glucose concentrations were measured using the ADVIA® 2400 chemistry system (Siemens, Bayern, Germany). Total glucose incremental area under the curve (iAUC) during OGTT was determined using the trapezoidal method [10]. Insulin concentration was measured using a Cobas e 601 module (Hitachi High-Technologies Corporation, Tokyo, Japan). C-peptide concentration was measured using a Cobas 8000 chemistry autoanalyzer (Roche Diagnostics System, Basel, Switzerland). Hemoglobin A1c (HbA1c) concentration was measured using an ADAMS A1C HA-8180 (Arkray Factory, Kyoto, Japan). Total cholesterol, triglyceride, HDL-cholesterol, and LDL-cholesterol concentrations were measured using a Hitachi 7600–110 analyzer (Hitachi, Tokyo, Japan).

### Safety outcome measures

At each visit, subjects were examined for electrocardiogram, vital signs (blood pressure and pulse rate), and laboratory tests (WBC, RBC, Hb, Hct, platelet count, ALP, γ-GT, AST, ALT, total bilirubin, total protein, albumin, BUN, and creatinine) for safety evaluation. WBC, RBC, Hb, Hct, and platelet count were measured using a System XE-5000TM (Sysmex, Kobe, Japan). ALP, γ-GT, AST, ALT, total bilirubin, total protein, albumin, BUN, and creatinine were measured using the ADVIA® 2400 chemistry system (Siemens, Munich, Germany).

### Evaluation of diet and physical activity

Dietary intake survey and physical activity survey were used to determine subjects’ lifestyle changes during the trial period. Dietary intakes investigated by the 3-day dietary record method were analyzed using the CAN-pro 4.0 program (The Korean Nutrition Society, Seoul, Korea). Physical activity was investigated and analyzed by Global Physical Activity Questionnaire (GPAQ).

### Statistical analysis

All statistical analyses were conducted using SAS version 9.4 (SAS Institute, Charlotte, NC, USA). Sample size was calculated to detect a 1.0 mg/dL (SD = 2.3 mg/dL) based on a power of 0.8 and an α levels of 0.05 [11]. Therefore, the calculated number of subjects was 21 for each group considering the dropout rate of 30%. Analysis was performed on a per protocol (PP) approach. Data are shown as the mean value and standard deviation (SD). To determine the differences between the groups, the categorized variables were analyzed using the chi-square test and continuous variables were analyzed using the *t*-test. A *p*-value less than 0.05 was considered statistically significant.

## Results

### Subject disposition

**Fig. 2:**
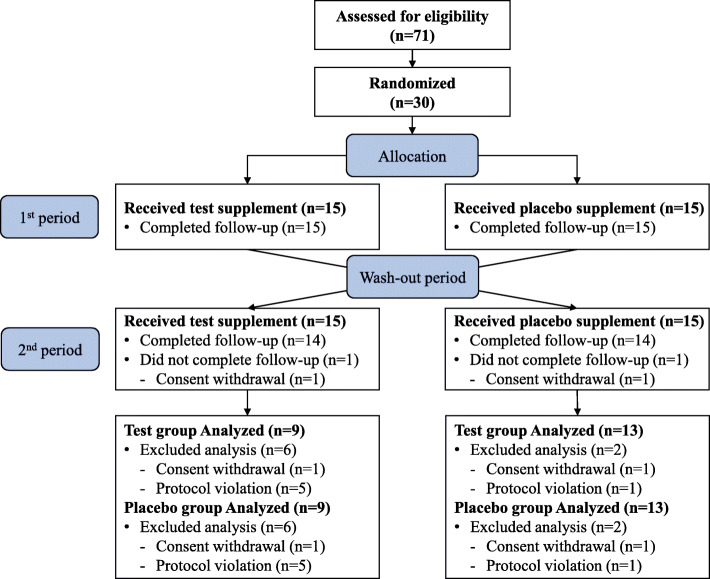
CONSORT diagram showing the study flow

Among the 30 subjects randomly assigned at study entry, 22 (73%) completed the study (Fig. 2).

### Subject characteristics

General characteristics of the subjects are shown in Table 2. Baseline characteristics of age, sex, weight, height, and FPG were not significantly different between the AHE and placebo groups.

**Table 2:** Demographic characteristics of the trial subjects^a^

Variable	Value
Sex (M/F)	13/17
Age (years)	54.17 ± 9.10
Height (cm)	162.33 ± 9.30
Weight (kg)	68.47 ± 13.05
BMI (kg/m^2^)	25.83 ± 3.04
FPG (mg/dL)	110.60 ± 10.22

*M* male; *F* female; *BMI* body mass index; *FPG* fasting plasma glucose

^a^Values are presented as number or mean ± standard deviation

### Safety evaluation

Thirteen adverse events were observed in three subjects in the AHE group and four in the placebo group during the trial period. The adverse events reported in the AHE group were common cold, back pain, skin peeling, dyspepsia, and upper respiratory tract infection. Adverse events were not significantly different between the two groups. There was no significant difference between the groups in the occurrence of adverse events, and it was judged that the adverse events had no relationship with AHE intake. Also, all safety parameter values, including ECG, vital signs, and laboratory tests, were within normal range during the trial period.

### Diet and physical activity

There were no significant differences between the groups in dietary intake (calories, carbohydrates, protein, fat, and fiber) or physical activity (metabolic equivalents value).

### Efficacy evaluation

The effects of AHE supplementation on plasma glucose level after OGTT and metabolic parameters were evaluated. A possible carryover effect for AHE supplementation was evaluated by comparing HbA1c levels at the end of the washout periods with baseline values. After 4-week washout period, there were no statistically significant differences in the HbA1c levels between placebo and AHE supplemented groups (data not shown), indicating no carryover effects of AHE supplementation.

After 8 weeks of supplementation, 1-h glucose level and iAUC were significantly different between the groups due to unexpected increases in the placebo group (*p* = 0.040 and *p* = 0.040, respectively) (Table 3). In addition, a significant difference was observed in the HbA1c level due to AHE supplementation (*p* = 0.012). Plasma levels of fasting glucose, insulin, C-peptide, total cholesterol, HDL-cholesterol, LDL-cholesterol, and triglyceride were not different between the AHE and placebo groups (Table 4).

**Table 3:** Blood glucose level obtained during the OGTT before and after supplementation with AHE or placebo^a^

		Test group (*n* = 22)			Placebo group (*n* = 22)			*P*-value^b^
		Week 0	Week 8	Change value	Week 0	Week 8	Change value	
Blood glucose (mg/dL)	0 min	106.91 ± 7.21	107.55 ± 10.66	0.64 ± 8.51	108.55 ± 9.77	107.77 ± 10.43	−0.77 ± 7.56	0.569
	30 min	182.36 ± 27.14	183.32 ± 20.41	0.95 ± 20.58	186.95 ± 28.25	187.64 ± 23.65	0.68 ± 27.52	0.968
	60 min	216.86 ± 32.74	216.18 ± 46.24	−0.68 ± 32.57	197.77 ± 39.54	216.27 ± 46.07	18.50 ± 33.20	0.040
	90 min	211.59 ± 50.02	209.27 ± 46.28	−2.32 ± 37.57	192.45 ± 44.93	214.45 ± 56.48	22.00 ± 55.40	0.149
	120 min	196.77 ± 48.95	192.32 ± 44.99	−4.45 ± 39.49	182.82 ± 47.79	195.09 ± 51.71	12.27 ± 47.65	0.282
iAUC_0–2 h_ (h*mg/dL)		167.51 ± 55.92	164.26 ± 57.42	−3.25 ± 38.59	144.37 ± 43.54	169.35 ± 63.80	24.98 ± 45.15	0.040

*OGTT* oral glucose tolerance test; *AHE Allium hookeri* extract; *iAUC* incremental area under the curve

^a^Values are presented as mean ± standard deviation

^b^Analyzed using *t*-test (difference between two groups in change from baseline)

**Table 4:** Blood markers obtained before and after supplementation with AHE or placebo^a^

	Test group (*n* = 22)			Placebo group (*n* = 22)			*P*-value^b^
	Week 0	Week 8	Change value	Week 0	Week 8	Change value	
Insulin (μU/mL)	8.52 ± 4.38	10.41 ± 5.00	1.89 ± 3.53	9.32 ± 4.42	9.26 ± 5.19	−0.06 ± 3.08	0.094
C-peptide (ng/mL)	2.00 ± 0.77	2.23 ± 0.69	0.23 ± 0.50	2.07 ± 0.77	2.18 ± 0.90	0.11 ± 0.38	0.448
HbA1c (%)	6.24 ± 0.38	6.20 ± 0.37	−0.04 ± 0.12	6.19 ± 0.37	6.26 ± 0.39	0.07 ± 0.15	0.012
Total cholesterol (mg/dL)	213.86 ± 27.18	212.68 ± 24.87	−1.18 ± 28.03	219.27 ± 21.88	217.68 ± 25.36	−1.59 ± 18.98	0.963
Triglycerides (mg/dL)	180.77 ± 117.66	170.09 ± 109.94	−10.68 ± 34.42	160.86 ± 75.71	157.32 ± 74.26	−3.55 ± 84.89	0.730
HDL-cholesterol (mg/dL)	49.27 ± 9.52	49.68 ± 8.25	0.41 ± 5.43	50.64 ± 9.42	48.45 ± 8.32	−2.18 ± 6.18	0.201
LDL-cholesterol (mg/dL)	133.86 ± 19.11	135.23 ± 19.86	1.36 ± 25.42	145.00 ± 18.97	143.82 ± 24.50	−1.18 ± 17.76	0.759

*AHE Allium hookeri* extract; *HbA1c* hemoglobin A1c

^a^Values are presented as mean ± standard deviation

^b^Analyzed using *t*-test (difference between two groups in change from baseline)

## Discussion

Genus *Allium*, such as garlic, and onion has various bioactive functions such as anti-cancer [12, 13], anti-inflammatory [14, 15], anti-diabetic [16, 17], and hypolipidemic [16, 18] effects, and these functions are known to be caused by sulfur compounds. Representative sulfur compounds include alliin, allicin, and cycloalliin. *A. hookeri* is a genus *Allium* containing cycloalliin as its main compound, and is reported to contain lower alliin, allicin and higher cycloalliin than other species [5]. Cycloalliin is a stable cyclic compound formed by sulfoxide such as alliin after processing such as crush and heat [19]. According to various reports, cycloalliin is also known to have antioxidant [5], hypolipidemic [20], and fibrinolytic [21] activities. Food can be consumed as raw materials, but it is common to undergo various cooking or processing, so it is important to keep the active compounds stable during processing. Therefore, *A. hookeri*, which contains a large amount of cycloalliin, a relatively stable active compound, has many advantages. For this reason, the AHE used in this trial were also standardized with cycloalliin as a marker compound.

This is the first clinical trial where the safety and efficacy of AHE in subjects with prediabetes were evaluated. AHE was well tolerated, and no serious adverse events occurred in the AHE group. Twice daily AHE supplementation resulted in decreased HbA1c level from baseline to 8 weeks compared with placebo. In addition, 1-h plasma glucose and iAUC showed significant differences between the groups, but this was due to unexpected increases of these parameters in the placebo group. Prediabetes has no specific signs or symptoms, however individuals with prediabetes are at increased risk of developing type 2 diabetes. Longitudinal studies to predict future diabetes development show that prediabetes subjects with a higher HbA1c level have a higher predictive value of progression to diabetes than those with a lower HbA1c level, although the results vary according to the population characteristics [22–24]. Because type 2 diabetes is a treatable but not curable disease, an approach to tackle or to delay this progression should be a superior health strategy than an approach to manage the disease after it is established. In this study, AHE supplementation has shown to be effective in reducing HbA1c level in prediabetes subjects. Given that HbA1c level reflects the average blood glucose levels over the past 3 months, it is tempting to speculate that AHE might be effective for long-term glycemic control, especially in ethnic subgroups who have a higher risk of type 2 diabetes progression. However, there is limitation in assessing the long-term efficacy of the test supplementation by measuring HbA1c, since the washout period is 4 weeks. The lack of a carryover effect for AHE supplementation based on HbA1c measurements allowed us to combine the data from the cross-over studies.

Overall, our findings are in agreement with previous cell culture and animal studies demonstrating anti-diabetic effects of AHE. Four studies have been conducted to investigate the underlying mechanism. First, AHE was shown to suppress body weight gain and organ fat mass [7]. Second, in a study with 3 T3-L1 adipocytes, AHE suppressed resistin secretion and increased secretion of adiponectin [25]. Third, AHE significantly reduced fasting blood glucose, HbA1c, and lipid profile and increased insulin in db/db mice [26, 27]. Finally, Rho et al. [9] showed that AHE-treated rats were protected from streptozotocin-induced pancreatic β-cell damage via suppression of the NF-κB signaling pathway. These in vitro and in vivo findings suggest that AHE supplementation may have anti-diabetic effects by maintaining insulin secreting capacity of pancreatic β-cells and by changing the secretion of adipocytokines involved in glucose metabolism.

After supplementation with AHE, statistically significant differences were found in plasma glucose level at an earlier time point during OGTT. These results suggest that AHE may slow digestion of ingested food in the intestinal lumen. Yang et al. [25] reported that AHE increases the expression of glucose transporter 4 (GLUT4) in 3 T3-L1 adipocytes, suggesting an additional possibility in which AHE improves glucose intolerance by increasing insulin action on the target tissues responsible for glucose uptake. Altogether, these findings indicate that, regardless of underlying mechanism, AHE supplementation is effective in reducing HbA1c level.

This study had several limitations that should be considered. First, this trial included a small number of subjects; therefore, caution should be used in generalizing the results to other populations. However, the results are generally consistent with the reported increase of glycemic responses by AHE in previous studies [9, 25]. Second, the subjects were allowed to maintain their usual diet and activities without conducting surveys regarding lifestyles. Therefore, the subjects’ diets and activity levels were not strictly controlled. For a more accurate study, control of lifestyle factors, such as food intake and physical activity, is necessary. Third, the short duration of the study (8 weeks) provided limited information on long-term anti-diabetic effects and/or on the long-term safety risks associated with AHE.

Studies on the anti-diabetic effects of the genus *Allium* containing abundant sulfur compounds with predominant biological functions have been continuously conducted. However, clinical trials for anti-diabetic effects in genus *Allium* such as garlic and onion has some limitations such as the fact that they are not RCT [28–30], do not confirm the significance compared to the placebo [31], or lack of data on postprandial glucose [17, 28, 30]. Despite several limitations, the trial, which used AHE containing sulfur compounds as a test product, conducted a well-designed RCT based on preclinical studies. Changes in various biomarkers related to blood glucose were observed for prediabetic subjects, and significant effects were shown in some items compared to the placebo group. Therefore, the findings of this trial confirmed the possibility of blood glucose improvement in AHE, which is the first-in-human and proof-of-concept clinical trial, so larger and longer trials are required to confirm the preliminary findings.

## Conclusion

In conclusion, 8 weeks after AHE supplementation, plasma glucose at 1-h OGTT, iAUC, and HbA1c level was significantly decreased in the AHE group compared with the placebo group. However, significant difference in 1-h plasma glucose level was determined by unexpected increase in placebo group. It is meaningful that this study was first study to evaluate the anti-diabetic effects of *A.hookeri* in humans. However, there is a limit to the small sample size of this study, so larger clinical trials are needed in the future.

## Supplementary information

**Additional file 1.** CONSORT 2010 checklist of information to include when reporting a randomised trial

## Data Availability

The datasets generated and/or analyzed during the study are not publicly available to protect subject confidentiality but are available from the corresponding author on reasonable request.

## Abbreviations

AHE: *Allium hookeri* extract
ALP: Alkaline phosphatase
ALT: Alanine transaminase
AST: Aspartate aminotransferase
BUN: Blood urea nitrogen
FPG: Fasting plasma glucose
γ-GT: Gamma-glutamyltransferase
GPAQ: Global Physical Activity Questionnaire
Hb: Hemoglobin
Hct: Hematocrit
HDL: High density lipoprotein
iAUC: Incremental area under the curve
LC-MS/MS: Liquid chromatography-mass spectrometry
LDL: Low density lipoprotein
OGTT: Oral glucose tolerance test
PP: Per protocol
RBC: Red blood cell
SD: Standard deviation
WBC: White blood cell

## References

[1] Forouhi NG, Wareham NJ (2014). Epidemiology of diabetes. Medicine (Abingdon).

[2] Chen L, Pei JH, Kuang J, Chen HM, Chen Z, Li ZW, Yang HZ (2015). Effect of lifestyle intervention in patients with type 2 diabetes: a meta-analysis. Metabolism.

[3] Association AD (2019). Lifestyle management: standards of medical care in diabetes-2019. Diabetes Care.

[4] Gupta P, Bala M, Gupta S, Dua A, Dabur R, Injeti E, Mittal A (2016). Efficacy and risk profile of anti-diabetic therapies: Conventional vs traditional drugs-A mechanistic revisit to understand their mode of action. Pharmacol Res.

[5] Kim S, Kim DB, Lee S, Park J, Shin D, Yoo M (2016). Profiling of organosulphur compounds using HPLC-PDA and GC/MS system and antioxidant activities in hooker chive (*Allium hookeri*). Nat Prod Res.

[6] Kim JE, Park KM, Lee SY, Seo JH, Yoon IS, Bae CS, Yoo JC, Bang MA, Cho SS, Park DH (2017). Anti-inflammatory effect of *Allium hookeri* on carrageenan-induced air pouch mouse model. PLoS One.

[7] Park S, No K, Lee J (2018). Anti-obesity effect of *Allium hookeri* leaf extract in high-fat diet-fed mice. J Med Food.

[8] Park H, Jeong J, Hyun H, Kim J, Kim H, Oh HI, Choi JY, Hwang HS, Oh DB, Kim JI (2016). Effects of a hot-water extract of *Allium hookeri* roots on bone formation in human osteoblast-like MG-63 cells *in vitro* and in rats *in vivo*. Planta Med.

[9] Roh SS, Kwon OJ, Yang JH, Kim YS, Lee SH, Jin JS, Jeon YD, Yokozawa T, Kim HJ (2016). *Allium hookeri* root protects oxidative stress-induced inflammatory responses and beta-cell damage in pancreas of streptozotocin-induced diabetic rats. BMC Complement Altern Med.

[10] Tai MM (1994). A mathematical model for the determination of total area under glucose tolerance and other metabolic curves. Diabetes Care.

[11] Kim HJ, Ahn HY, Kwak JH, Shin DY, Kwon YI, Oh CG, Lee JH (2014). The effects of chitosan oligosaccharide (GO2KA1) supplementation on glucose control in subjects with prediabetes. Food Funct.

[12] Ishikawa H, Saeki T, Otani T, Suzuki T, Shimozuma K, Nishino H, Fukuda S, Morimoto K (2006). Aged garlic extract prevents a decline of NK cell number and activity in patients with advanced cancer. J Nutr.

[13] Miraghajani M, Rafie N, Hajianfar H, Larijani B, Azadbakht L (2018). Aged garlic and Cancer: a systematic review. Int J Prev Med.

[14] Darooghegi Mofrad M, Milajerdi A, Koohdani F, Surkan PJ, Azadbakht L (2019). Garlic supplementation reduces circulating C-reactive protein, tumor necrosis factor, and Interleukin-6 in adults: a systematic review and meta-analysis of randomized controlled trials. J Nutr.

[15] Xu C, Mathews AE, Rodrigues C, Eudy BJ, Rowe CA, O'Donoughue A, Percival SS (2018). Aged garlic extract supplementation modifies inflammation and immunity of adults with obesity: a randomized, double-blind, placebo-controlled clinical trial. Clin Nutr ESPEN.

[16] Shabani E, Sayemiri K, Mohammadpour M (2019). The effect of garlic on lipid profile and glucose parameters in diabetic patients: a systematic review and meta-analysis. Prim Care Diabetes.

[17] Sobenin IA, Nedosugova LV, Filatova LV, Balabolkin MI, Gorchakova TV, Orekhov AN (2008). Metabolic effects of time-released garlic powder tablets in type 2 diabetes mellitus: the results of double-blinded placebo-controlled study. Acta Diabetol.

[18] Jung ES, Park SH, Choi EK, Ryu BH, Park BH, Kim DS, Kim YG, Chae SW (2014). Reduction of blood lipid parameters by a 12-wk supplementation of aged black garlic: a randomized controlled trial. Nutrition.

[19] Lee HJ, Suh HJ, Han SH, Hong J, Choi HS (2016). Optimization of extraction of Cycloalliin from garlic (Allium sativum L.) by using principal components analysis. Prev Nutr Food Sci.

[20] Yanagita T, Han SY, Wang YM, Tsuruta Y, Anno T (2003). Cycloalliin, a cyclic sulfur imino acid, reduces serum triacylglycerol in rats. Nutrition.

[21] Agarwal RK, Dewar HA, Newell DJ, Das B (1977). Controlled trial of the effect of cycloalliin on the fibrinolytic activity of venous blood. Atherosclerosis.

[22] Bae JC, Rhee EJ, Lee WY, Park SE, Park CY, Oh KW, Park SW, Kim SW (2011). Optimal range of HbA1c for the prediction of future diabetes: a 4-year longitudinal study. Diabetes Res Clin Pract.

[23] Bonora E, Kiechl S, Mayr A, Zoppini G, Targher G, Bonadonna RC, Willeit J (2011). High-normal HbA1c is a strong predictor of type 2 diabetes in the general population. Diabetes Care.

[24] Liang K, Wang C, Yan F, Wang L, He T, Zhang X, Li C, Yang W, Ma Z, Ma A (2018). HbA1c Cutoff Point of 5.9% Better Identifies High Risk of Progression to Diabetes among Chinese Adults: Results from a Retrospective Cohort Study. J Diabetes Res.

[25] Yang HS, Choi YJ, Jin HY, Lee SC, Huh CK (2016). Effects of *Allium hookeri* root water extracts on inhibition of adipogenesis and GLUT-4 expression in 3T3-L1 adipocytes. Food Sci Biotechnol.

[26] Kim NS, Choi BK, Lee SH, Jang HH, Kim JB, Kim HR, Choe JS, Cho YS, H YJ, Kim YS (2016). Effects of Allium hookeri extracts on glucose metabolism in type II diabetic mice. Kor J Pharmacogn.

[27] Lee SH, Kim NS, Choi BK, Jang HH, Kim JB, Lee YM, Kim DK, Lee CH, Kim YS, H YJ (2015). Effects of Allium hookeri on lipid metabolism in type II diabetic mice. Kor J Pharmacogn.

[28] Choudhary PR, Jani RD, Sharma MS (2018). Effect of raw crushed garlic (Allium sativum L.) on components of metabolic syndrome. J Diet Suppl.

[29] Kumar R, Chhatwal S, Arora S, Sharma S, Singh J, Singh N, Bhandari V, Khurana A (2013). Antihyperglycemic, antihyperlipidemic, anti-inflammatory and adenosine deaminase- lowering effects of garlic in patients with type 2 diabetes mellitus with obesity. Diabetes Metab Syndr Obes.

[30] Mahmoodi M, Islami MR, Asadi Karam GR, Khaksari M, Sahebghadam Lotfi A, Hajizadeh MR, Mirzaee MR (2006). Study of the effects of raw garlic consumption on the level of lipids and other blood biochemical factors in hyperlipidemic individuals. Pak J Pharm Sci.

[31] Jafarpour-Sadegh F, Montazeri V, Adili A, Esfehani A, Rashidi MR, Pirouzpanah S (2017). Consumption of fresh yellow onion ameliorates hyperglycemia and insulin resistance in breast Cancer patients during doxorubicin-based chemotherapy: a randomized controlled clinical trial. Integr Cancer Ther.

